# Estimating changes in flood risks and benefits of non-structural adaptation strategies - a case study from Tyrol, Austria

**DOI:** 10.1007/s11027-014-9602-3

**Published:** 2014-10-31

**Authors:** Annegret H. Thieken, Holger Cammerer, Christian Dobler, Johannes Lammel, Fritz Schöberl

**Affiliations:** 1 Institute of Earth and Environmental Sciences, University of Potsdam, Karl-Liebknecht-Str. 24-25, 14476 Potsdam, Germany; 2 Institute of Geography, University of Innsbruck, Innrain 52f, 6020 Innsbruck, Austria; 3 alpS GmbH, Grabenweg 68, 6020 Innsbruck, Austria; 4 Present Address: DEVK Rückversicherungs- und Beteiligungs-AG, Riehler Str. 190, 50735 Cologne, Germany; 5 Present Address: Government of Tyrol, Heiliggeiststraße 7-9, 6020 Innsbruck, Austria; 6 Present Address: TIWAG-Tiroler Wasserkraft AG, Eduard-Wallnöfer-Platz 2, 6020 Innsbruck, Austria

**Keywords:** Flood risk, Scenarios, Adaptation to climate change, Hazard, Vulnerability, Lech catchment

## Abstract

Flood damage has increased significantly and is expected to rise further in many parts of the world. For assessing potential changes in flood risk, this paper presents an integrated model chain quantifying flood hazards and losses while considering climate and land use changes. In the case study region, risk estimates for the present and the near future illustrate that changes in flood risk by 2030 are relatively low compared to historic periods. While the impact of climate change on the flood hazard and risk by 2030 is slight or negligible, strong urbanisation associated with economic growth contributes to a remarkable increase in flood risk. Therefore, it is recommended to frequently consider land use scenarios and economic developments when assessing future flood risks. Further, an adapted and sustainable risk management is necessary to encounter rising flood losses, in which non-structural measures are becoming more and more important. The case study demonstrates that adaptation by non-structural measures such as stricter land use regulations or enhancement of private precaution is capable of reducing flood risk by around 30 %. Ignoring flood risks, in contrast, always leads to further increasing losses—with our assumptions by 17 %. These findings underline that private precaution and land use regulation could be taken into account as low cost adaptation strategies to global climate change in many flood prone areas. Since such measures reduce flood risk regardless of climate or land use changes, they can also be recommended as no-regret measures.

## Introduction

Of all natural hazards, floods are responsible for the largest economic losses worldwide (Munich Re 1997, 2004). In the future, climate change may contribute to an increase in flood losses in several regions due to an augmentation of flood frequencies and magnitudes. However, the impact of climate change differs from catchment to catchment (Smith 1999; Schreider et al. 2000; Hall et al. 2005) and up to now there is no clear signal of trends in river floods (see e.g. Svensson et al. 2006 or Blöschl et al. 2011 for discussion). Hence, the Intergovernmental Panel on Climate Change (IPCC 2012) finds only limited to medium evidence for observed changes in the magnitude and frequency of floods at regional scales due to limited flood records and confounding effects of changes in land use and engineering. Further, projections of changes in flooding are of low confidence, due to the complexity of regional changes in meteorology and hydrology (IPCC 2012).

In fact, human-induced changes in land use have been identified to play a key role in flood risk development (e.g. Hall et al. 2006; Feyen et al. 2009; Merz et al. 2010a; Beckers et al. 2013; Jongman et al. 2014). Ongoing settlement and economic development have led to a continuous increase in assets in flood prone areas. In developing countries, this trend is due to population growth. In industrialised countries, comparatively low prices for building land, good transportation infrastructure and the proximity to cities serve as further explanations. In Europe, growing losses have not only been attributed to increasing population, but also to wealth and inflation (Barredo 2009). Consequently, urban and spatial planning are crucial for the development of flood risks (White and Howe 2002; Petrow et al. 2006) and have to be regarded when future flood risks are to be estimated. However, climate impact studies on flooding that also include vulnerability and risk aspects are still rare. Only a few attempts have been made to assess flood risk by integrating possible future changes in both, climate and socio-economic development (e.g. Hall et al. 2005; te Linde et al. 2011; Elmer et al. 2012; Beckers et al. 2013; Jongman et al. 2014). In this paper, changes in land use, economic development and climate were therefore combined to achieve a more holistic, future-oriented flood risk analysis.

In the context of this paper, flood risk is defined as the product of hazard, i.e. the physical and statistical aspects of the flooding process (e.g. return period of the flood, extent and depth of inundation), and vulnerability, i.e. the exposure of people and assets to floods and the susceptibility of elements at risk of suffering flood damage (e.g. Mileti 1999; Merz and Thieken 2004). In risk analyses with a technical focus, vulnerability comprises two elements (Merz and Thieken 2004; Kron 2005): 1) the asset values at risk, i.e. the buildings, infrastructures, humans etc. that are exposed to flooding (inundation); and 2) the susceptibility of the exposed structures, e.g. the lack of resistance against damaging/destructive forces. Thus flood risk can be defined as: Risk = Hazard x Values at Risk x Susceptibility. Changes in flood risks can hence be attributed to – but can also be governed by – changes in flood hazard, elements at risk (exposure) or their susceptibility to flooding.

Mountain regions like the European Alps are particularly prone to different kinds of natural hazards such as all types of mass movements and floods. From 1980 to 2005, about two thirds of all economic losses due to natural hazards in the European Alps were caused by floods (OECD 2007). In Austria, for example, recent flood events in May 1999, August 2002, August 2005 as well as in June 2013 caused damage of 35 Million Euro, 2,445 Million Euro, 515 Million Euro (Munich Re 2007) and 866 Million Euro (as at 6 August 2013; EC 2013), respectively. These events indicate the considerable hazard and damage potential of natural events in the Alpine space (BMLFUW 2006; Stötter 2007).

Pfurtscheller et al. (2011) mention four features that make Alpine areas and other mountain regions especially vulnerable to natural hazards: i) intermixtures of hazards, e.g. flooding, debris/mud flows and landslides, leading to severe damage, ii) limitation of permanent settlement areas and missing possibilities to relocate settlements, lifelines and transport networks, iii) special situation of lateral valleys with limited accessibility, and iv) monosectorality of Alpine economies, e.g. on tourism, and high mobility of manpower, which results, e.g., in commuting traffic.

Due to the topography of Alpine areas, only 17 % of the total area of the European Alps is suitable for permanent settlement (Tappeiner et al. 2008), infrastructures and lifelines. The high concentration of people and assets in the valleys is reflected by the high population density of 400 people per square kilometre in areas of permanent settlement, which are still at risk in some places. For example, in the municipality of Ischgl, Federal state of Tyrol, Austria, nearly 75 % of the whole permanent settlement area is threatened by flood, debris flow and/or avalanche events with return periods of 150 years (Pfurtscheller et al. 2011). The hazard situation of lateral valleys needs special attention since they are often accessible by only one lifeline. Therefore, enormous efforts are undertaken to protect lifelines, e.g. by the construction of avalanche sheds, also in remote areas with only few inhabitants (Pfurtscheller et al. 2011). Although the most recent flood event in June 2013 again caused tremendous damage, it has to be acknowledged that existing protection measures vastly reduced damage (Blöschl et al. 2013).

Furthermore, mountain regions are particularly vulnerable to climate change (e.g. Beniston 2003). In the European Alps, the increase in temperature between 1906 and 2005 was twice as high as the global average (Brunetti et al. 2009). With regard to precipitation, increasing amounts in winter as well as higher frequency of heavy precipitation events have been observed in past decades (Widmann and Schär 1997; Frei and Schär 2001). There are already some indications for changing flood frequencies and magnitudes in the Swiss Alps (Allamano et al. 2009; Schmocker-Fackel and Naef 2010). In Austria, different regional trends were identified (Blöschl et al. 2011).

In order to investigate trends of future floods, scenario analyses have often been used, but include high uncertainties. Since outputs of general circulation models (GCMs) are too coarse to allow statements on the regional scale, downscaling methods, i.e. regional circulation models (RCMs) or statistical downscaling methods, are needed. In regions with a complex topography, like the European Alps, however, the spatial resolution of RCMs still do not allow to investigate local processes (Engen-Skaugen 2007). Systematic biases, especially in the simulation of precipitation, have been reported for the Alps (e.g. Frei et al. 2006; Smiatek et al. 2009; Themeßl et al. 2010; 2012). Therefore, climate change impact studies on floods in alpine regions are still a challenge. With regard to future land use developments in risk assessments, alpine studies focused only on other hazard types like landslides (e.g. Promper and Glade 2012) or glacier lake outburst floods (e.g. Nussbaumer et al. 2014), but not on river floods so far.

The objectives of this paper are twofold. First, the paper aims at quantifying changes in flood risks in an alpine environment between 2006 and 2030 by setting up and adapting a model chain that accounts not only for climate change impacts, but also for changes in land use and exposed asset values (economic development). It is investigated to which degree changes in flood risks in an alpine catchment can be attributed to climate change, land use change or economic development. This should lead to recommendations with regard to the general set-up of climate impact studies on flood risks.

Second, the impacts of different adaptation options on the flood risk are analysed in order to derive recommendations for adaptation options to global climate change. Special emphasis is placed on non-structural, low cost adaptation measures like the improvement of the precautionary behaviour of residents or stricter land use regulations. Using the Tyrol, Austria Upper Lech catchment as an example, the potential of such measures for damage reduction will be explored on the regional scale, which is particularly relevant for the development and implementation of adaptation strategies.

## Investigation area

**Fig. 1:**
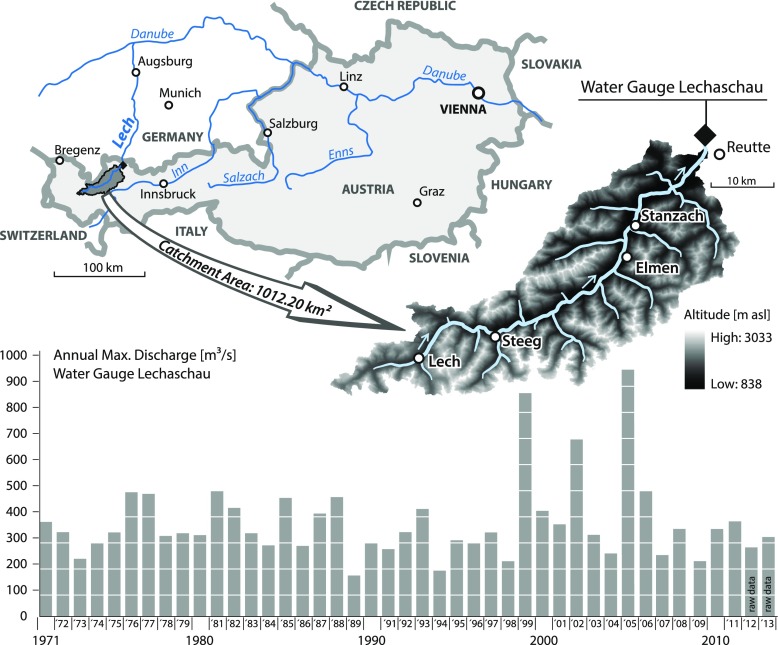
Location of the investigation area and series of annual maximum discharges at the outlet water gauge Lechaschau

As investigation area, the upper part of the catchment area of the river Lech with particular emphasis on the area around Reutte in Tyrol, Austria, was chosen. The catchment has an area of 1,012 km^2^ and covers around one quarter of the whole Lech watershed up to Marxheim in Germany (see Fig. 1), where it discharges into the river Danube. The catchment can be described as a typical Alpine valley with high topography and steep slopes: altitude ranges from 838 m above sea level at the outlet of the basin to 3,038 m above sea level at the highest mountain peak. 13 weather stations with daily data on temperature and precipitation covering at least the period from 1989 to 2005 are located within the catchment or in close vicinity and were used for this study.

Based on observations from 1971 to 2005, the annual precipitation varies strongly within the catchment from around 1,300 to 1,800 mm, with a maximum monthly precipitation of 172 mm in July (Dobler et al. 2013). Since precipitation between November and March mostly falls as snow (Dobler et al. 2010), the catchment is characterised by a nivo-pluvial runoff regime, with a minimum runoff observed during winter and a maximum runoff in late spring and summer.

Daily runoff has been measured at the outlet gauge at Lechaschau since 1971. Long-term mean daily runoff at the outlet gauge at Lechaschau is approximately 45 m^3^/s. Major floods can be caused either by a combination of snowmelt and heavy rainfall or by heavy rainfall alone. Recent severe flooding occurred in 1999, 2002 and 2005 with peak discharges of 855, 676 and 943 m^3^/s, respectively. In June 2013, when many regions in Central Europe were flood-affected, most of the precipitation in the study area was stored as snow; hence, the peak discharge was not very high (see raw data in Fig. 1).

The area of Reutte, where the population density is highest within the Austrian part of the Lech catchment, is characterised by strong socio-economic dynamics in the past due to an increase in the population (e.g. 50 % between 1961 and 2001), migration and commuter balance (Amt der Tiroler Landesregierung 2008). It offers most of the workplaces in the Austrian Lech Valley, mainly in the service sector as well as in the industrial sector, i.e. metal and wood working. Agriculture decreased drastically in the last decades (Amt der Tiroler Landesregierung 2008), which is typical for the shift from a traditionally agricultural society to a service-, industry- and leisure-oriented society in the Alpine space (Bätzing 2003; Holub and Fuchs 2009).

## Data and methods

### Model chain

**Fig. 2:**
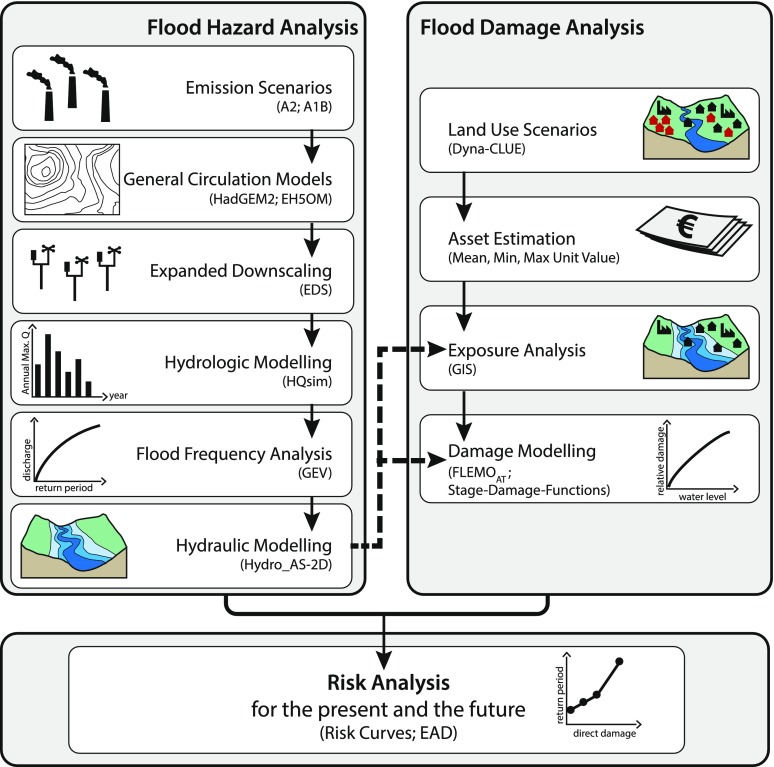
Elements of the model chain that was applied to assess present and future flood risks in the study area; models and abbreviations are explained in the section Data and Methods

The study was designed as a pilot project for flood risk analyses that accounts for changes in climate and land use in an Alpine region. Following the above-mentioned definition of risk, meteorological, hydrological and hydraulic investigations to quantify the flood hazard as well as estimations of flood losses to characterise the societal vulnerability were undertaken separately (see Fig. 2). The flood hazard analysis included:

i. downscaling of general circulation model (GCM) results by the expanded downscaling (EDS) technique introduced by Bürger (1996),
ii. hydrological modelling with the conceptual semi-distributed rainfall—runoff model HQsim (that is an abbreviation of the German expression for simulated flood discharge) (Dobler and Pappenberger 2013),
iii. flood frequency analysis by the generalised extreme value (GEV) distribution, and
iv. hydrodynamic modelling to assess changes in inundated areas by the two-dimensional model Hydro_AS-2D (Nujic 2003).

The socio-economic investigations consisted of:

i. land use modelling with Dyna-CLUE, the DYNAmic and spatially explicit model for the Conversion of Land Use and its Effects (Verburg and Overmars 2009),
ii. identifying flood-exposed assets at risk by spatial analyses with ArcGIS, the Geographic Information System (GIS) of the Environmental Systems Research Institute (ESRI), Redlands, CA, USA, and
iii. assessing the vulnerability to quantify changes in the damage potential by using different loss models (Cammerer et al. 2013a).

Finally, flood probabilities and vulnerability assessments were combined to estimate the risk for present and future conditions. The single components of the model chain are briefly explained in the following sections. Results of the calibration and validation procedures can be found in the section Calibration and validation of the model chain.

#### Regional climate change scenarios – downscaling GCM results

As first part of the model chain, impacts of climate change had to be derived for the weather gauging stations in the study area. Therefore, the output of general circulation models (GCMs) was downscaled to a finer spatial resolution, with a special focus on reproducing extreme weather events.

Since regional circulation models (RCMs) were found to deliver biased results in the Alps (see introduction), the technique, introduced by Bürger (1996) was applied. EDS is a statistical downscaling technique which belongs to the group of regression methods. Thereby, a linear function between the large-scale variables (predictors) and the local-scale variables (predictands) is established. EDS is largely based on the concept of a multiple linear regression (MLR), which is frequently applied in statistical downscaling of GCM output (e.g. Maraun et al. 2010). However, the least-squares criterion of MLR significantly reduces the variability of local climate variables. Therefore, Bürger (1996) modified this concept in order to better simulate weather extremes. A side condition was added to the MLR definition which expresses the preservation of the local covariance. A full description of the EDS model is given in Bürger et al. (2009). Further details on the application in the Lech area are described in Dobler et al. (2013).

ECMWF-interim data (ECMWF stands for European Centre for Medium-Range Weather Forecasts) were used to calibrate and validate the EDS model for the Lech area, based on data from 1989 to 2000 and from 2001 to 2005, respectively. The calibrated EDS model was then used to downscale the output of GCMs (control and scenario runs). In order to assess and to reduce possible uncertainties involved in the climate projections a set of different climate models and scenarios was used. The output of two GCMs was downscaled to a finer spatial resolution. The GCMs were 1) EH5OM, the fifth generation of the global climate model ECHAM5, in which EC stands for ECMWF and HAM for Hamburg, Germany, where the model was developed at the Max Planck Institute (MPI) for Meteorology, coupled with MPI’s oceanic model (OM), and 2) HadGEM2 (Hadley Centre Global Environmental Model, version 2), a coupled Earth System Model being used by the Met Office Hadley Centre, United Kingdom.

The EH5OM simulation run was based on the A2-scenario as defined in the Special Report on Emission Scenarios (SRES), while the HadGEM2 simulation run was forced by the A1B-scenario. While the A1B-scenario is based on a rapid economic growth with, however, a rapid introduction of new and more efficient technologies that leads to a reduction of regional differences and per capita income, the A2-scenario assumes a heterogeneous world with preservation of local identities and a regionally oriented development. In terms of global greenhouse gas emissions, the A1B-scenario is a mid-range scenario, while the A2-scenario represents the upper bound.

Three ensemble integrations of EH5OM were used for the control run and the future scenario. The HadGEM2 simulations included one simulation for the control run and three ensemble integrations for the future scenario. The ensemble members of each GCM were treated as one 90-year experiment for the present and future scenario as suggested by Frei et al. (2006). For this study, the time slices from 1971 to 2000 were considered as reference period, those from 2016 to 2045 as future scenario.

#### Hydrological modelling

The hydrological model HQsim was used to simulate the hydrological behaviour of the Lech basin at the gauge Lechaschau. HQsim is a conceptual semi-distributed rainfall–runoff model, which was specially designed for the simulation of runoff in mountainous watersheds. The model is largely based on the water balance model developed by Federer and Lash (1978). Details of the model are reported in Dobler and Pappenberger (2013).

In a first step, a sensitivity analysis was performed with the HQsim model. Sensitivity analysis is an important tool i) to better understand how these complex models work, ii) to verify the model structure, and iii) to determine the key parameters, which exhibit major influence on the simulation results (Sieber and Uhlenbrook 2005; Manache and Melching 2008). The latter is of particular interest in order to reduce the dimensionality of the parameter space, which is important for a variety of applications, such as model calibration, parameter estimation or uncertainty assessment in order to identify the most important parameters of the complex model. In this study, three different sensitivity analysis techniques were applied, namely i) regional sensitivity analysis (RSA), ii) Morris analysis and iii) state dependent parameter (SDP) Modelling.

In a next step, the HQsim model was calibrated and validated based on observed meteorological and hydrological data. The time series from 1989 to 2005 was split into a calibration (1989 to 2000) and a validation (2001 to 2005) period. It should be noted that the chosen periods are identical to those used for calibrating and validating the EDS model. In a final step, the performance of the modelling chain consisting of the EDS and HQsim models was tested in reproducing observed runoff data at Lechaschau using ECMWF data as input.

After model calibration and validation, the downscaled output of the two GCMs, i.e. EH5OM and HadGEM2, was used to force the hydrological model in order to obtain runoff series for present and future climate conditions.

#### Flood frequency analysis

Observed and simulated discharge time series were used to estimate the frequency-magnitude-relationship of floods. Estimating the frequency of extreme floods is a key element, but also a major challenge in flood risk analysis. A robust estimation of extreme events requires long flood records in order to reliably extrapolate long return periods (e.g. Merz and Thieken 2009). However, in Alpine catchments long-term measurements are often missing. This hampers the application of flood frequency analysis if observed runoff data has to be used as input. In this study, the generalized extreme value (GEV) distribution was applied to different annual maximum discharge series derived from discharge measurements at the gauge Lechaschau. In order to reduce uncertainty, information on a historic flood event in 1910, which was measured at the downstream gauge at Füssen (Germany) and reported for the Upper Lech Valley by Meier (2002), was included by using the procedure of DVWK (1999), i.e. the data gap between the historical event in 1910 and the beginning of continuous measurements in 1971 was filled several times with observed flood discharges that fall below the discharge attributed to the historical event (see Thieken et al. 2011). This is based on the assumption that in the data gap the statistical characteristics of the observed time series are also valid (DVWK 1999; Merz and Thieken 2009).

A GEV-distribution was fitted to the series of annual discharge maxima of both the control and scenario simulations produced by the model chain, i.e. emission scenario – GCM – EDS – HQsim. Relative changes in the flood peak flows at certain return intervals, i.e. 30, 100, 200 and 300 years, between the control and scenario simulations were calculated and the changes were superimposed to the corresponding peak flows that were derived from the observed series with historic flood records. For the original and altered peak discharges that correspond to the return periods 30, 100, 200 and 300 years, hydrodynamic simulations were performed.

#### Hydrodynamic modelling

While the hydro-meteorological modelling was performed for the entire upper catchment of the river Lech, the impact analysis that mainly consisted of hydrodynamic simulations and loss estimations was restricted to the area of Reutte, which is the most important settlement area in the investigation area.

The two-dimensional hydrodynamic model Hydro_AS-2D (Nujic 2003) was applied in this study. This model has been applied as a standard system for flood routing in Bavaria, Germany, the neighbouring region to our study area (Dorner et al. 2008) and also for hydraulic scenarios in an Alpine foreland river in Austria (e.g. Neuhold 2013). The spatial discretisation is based on the finite-volume method, whereas the temporal discretisation is solved by the Runge–Kutta method.

Pre- and post-processing of the two- and three-dimensional finite elements was carried out by means of the Surface-water Modelling Solution Software (SMS) from Aquaveo™ (http://www.aquaveo.com). Thereby, a mesh of the river channel at a length of 10 km with its embankments was derived integrating 40 cross section profiles of the Lech River and laser scanning data with 1 m horizontal resolution from the Tyrolean government. In the hinterland of the levees a flood plain model was built by means of the same laser scanning data and the official building map. Furthermore, hydraulic relevant structures like bridges and structural protection measures, e.g. flood walls at the municipality of Lechaschau, were considered in the terrain model.

Roughness coefficients for the flood plain were derived for different land use classes of the current land use map from the government and for different parts of the river channel according to the Manning/Strickler formula. The hydrologic boundary conditions were adjusted for the inlet discharge according to the discharge at the gauge Lechaschau located close to the district capital Reutte (see Fig. 1). The 2D-simulations were performed with the peak discharge observed in August 2005 and the structural measures that were in place at that time. Subsequently, simulations with peak discharges that correspond to different recurrence intervals (30-year, 100-year, 200-year and 300-year) for the present as well as the future situation were performed considering the most recent flood control measures, e.g. heightening of the embankments after the flood in 2005. For all simulations, the flood wave observed in August 2005 was used and scaled by the peak discharge that corresponds to the peak flow of the scenario under study.

The resulting simulated maximum water depths (in m above ground surface) and water levels (in m above sea level) were provided as 1 m grid. For the intersection with the asset values and the assessment of flood damage (see below) the maximum water depths were aggregated to a cell size of 10 m by using the mean of the input cells.

#### Land use scenarios

With this component of the model chain, it was aimed at adopting a GIS-based algorithm that is capable of generating realistic land use changes for the region around the city of Reutte. Among the variety of land use models, the spatially explicit land use model on ‘Conversion of Land Use and its Effects’ CLUE (Verburg et al. 2002), in particular its adapted and dynamic version Dyna-CLUE (Verburg and Overmars 2009), was applied to simulate future land use patterns in the area of Reutte (see Cammerer et al. 2013b). The newer version Dyna-CLUE (Verburg and Overmars 2009) combines the top-down allocation of land use change to grid cells with a bottom-up determination of conversions for specific land use transitions. The spatially explicit allocation module allocates the lumped regional demands to individual grid cells by an iterative procedure.

A substantial prerequisite for all land use models is land use/cover information that represents the actual land use/cover at a certain point in time. As existing databases provided only coarse information, a new land use dataset that represents the year 2006 was created from digital colour orthophotos from an aerial survey in 2005/2006 (see Cammerer et al. 2013b). In total, nine land use classes were derived and converted into a raster format with a horizontal resolution of 50 m. This dataset was used to calibrate Dyna-CLUE. Thereby, a variety of potential land use drivers was considered in the statistical model (logistic regression), i.e. 1) socio-economic parameters (e.g. population density), 2) biophysical data (e.g. climate, topography), 3) accessibility parameters (e.g. distance to town centre or street), and 4) neighbourhood interactions. For indicating the goodness of fit of the logistic regression model the relative operating characteristic (ROC) method (Swets 1988) was used.

For the future scenario generation the land use demand of the different land use types had to be assessed. In this study, the four national spatial planning scenarios for Austria until 2030, published by the Austrian Conference on Spatial Planning (*Österreichische RaumOrdnungsKonferenz* - ÖROK 2008), were used. The four integrated spatial development scenarios of the ÖROK are based on a participatory approach calling for the input of multiple stakeholders, and they are not constrained by existing policies (Williams et al. 2009). The four ÖROK land use scenarios are in brief (see ÖROK 2008; Cammerer et al. 2013b):

- Overall Growth: Population, economy, tourism, transport and mobility are assumed to grow intensively, which ultimately results in a high demand for building land.
- Overall Competition: Also in this scenario, population, economy, tourism and transport grow intensively, but it is assumed that the market reacts timely to scarcities in order to avoid energy and environmental crises. Spatial development and pressure differ regionally.
- Overall Security: In this scenario only a moderate growth is assumed. In the favoured agricultural and forestry used regions, the spatial pressure increases due to the higher demand for biomass energy. Higher costs for mobility are in favour of urban agglomerations and centres.
- Overall Risk: Structural developments are comparable to the scenario ‘Overall Competition’. There is, however, a lack of mechanisms against sudden energy scarcity. Since spatial development in Austria is mainly forced by high energy and mobility costs, spatial development in this scenario is determined by the agglomeration of built-up areas and the exploitation of natural resources for energy production.

Besides these scenarios that determine the overall land use demand, spatial policies like building restrictions in hazard zones or in NATURA2000 areas, a European network of protected habitats, were included in the scenario generation with Dyna-CLUE, as they may constrain or prefer (in case of the area zoning plan) specific developments.

#### Asset estimation

All elements in the investigation area Reutte that are potentially at risk of being flooded had to be evaluated on an economic basis, i.e. a representative monetary value had to be assigned to each land use type. In general, assets can be divided in monetary, tangible and intangible assets, from which only tangible assets are usually of interest for damage assessment studies (Meyer 2005). Furthermore, the assessment concept has to be defined, i.e. the usage of replacement values or depreciated values (Meyer 2005; Messner et al. 2007; Merz et al. 2010b). In this study, replacement values were applied, which assume that damaged properties (or damaged parts of a property) will be replaced by new, similar structures. Replacement values are commonly used for assessments in the (re-)insurance sector.

As a basis, we used the aggregated replacement values for each municipality in the investigation area from the database of Huttenlau and Stötter (2008). In that study, replacement values for the year 2006 were distinguished for six functional classes, i.e. residential, mixed usage, agriculture, open land, industry and commerce as well as tourism. After assigning all land use types of our study area to one of these functional classes, specific values (in €/m^2^) could be derived by dividing the aggregated building values by the residential area of each municipality in 2006. For all subsequent analyses an average replacement value for buildings (€ 279 per m^2^) was used for the whole study area. Moreover, the upper and lower limits that were found in the study area were used as uncertainty measure (see Cammerer and Thieken 2013).

The asset values for the future land use scenarios were estimated by means of two different concepts. On the one hand, for each scenario and all points in time, constant values, i.e. asset values at constant prices of the reference year 2006, were assigned to all grid cells occupied by residential areas to discount for inflation. On the other hand, adjusted values were calculated to account for changes in the economic values themselves, which is often neglected in risk assessments for the future (e.g. Feyen et al. 2009; te Linde et al. 2011). As pointed out by Bouwer (2013), the total increase in asset values consists of new assets due to land use change, e.g. by new residential areas, as well as appreciation of existing values by technical innovation, improvement or maintenance and repair. Therefore, we introduced an additional correction factor by means of the gross domestic product (GDP) as an approximate indicator of the value development over time following the approach of Bouwer et al. (2010) and de Moel et al. (2011). The mean annual average growth rate of the GDP was published together with the ÖROK scenarios (ÖROK 2008) and could therefore be surcharged by means of a correction factor. According to Bouwer et al. (2010), the GDP had to be further corrected by relative changes of allocated built-up areas, since economic growth is already partly covered by the expansion of built-up areas that are simulated by the land use model. Finally, all grid cells occupied by residential areas in the different land use scenarios obtained an adjusted specific building replacement value, expressed in prices of the reference year 2006 (for further details see Cammerer and Thieken 2013).

For deriving assets at risk, the total building asset values for each point in time and for both concepts, i.e. constant values and adjusted values, were intersected with the four different inundation scenarios, i.e. the 30-, 100-, 200- and 300-year flood.

#### Damage estimation

Flood losses are commonly classified in direct and indirect damage (e.g. Smith and Ward 1998; Merz et al. 2010b). In this study, we limit the estimation of flood losses to direct, structural flood damage of residential buildings. For this, the inundation scenarios and the land use scenarios with assigned building asset values were combined by means of flood loss models. Commonly, this is done by relative depth-damage functions, in which the percental damage of the affected building is given in relation to the water level at that building.

To derive empirical functions, flood loss data have to be collected in the aftermath of a flood event (Merz et al. 2010b; Thieken et al. 2010). In Austria, flood loss data are generally collected in the frame of compensation payments by the Austrian disaster funds, but do not contain the relevant information that allow to relate a flood loss to a certain water depth (Habersack et al. 2004). For our study, aggregated loss data for the flood in August 2005 in the study region were used to validate different flood loss models as shown by Cammerer et al. (2013a). For the derivation of flood loss models, we relied, however, on comprehensive flood loss data that were collected in the aftermath of flood events in Germany in 2002, 2005 and 2006. Two surveys with computer-aided telephone interviews were carried out in 2003 and 2006, respectively, among flood affected private households. Besides flood losses in the residential sector, potential flood damage influencing factors like water depth, flood duration, contamination, precautionary and emergency measures were collected (for a more detailed description of these campaigns see Thieken et al. 2005; Kreibich et al. 2011; Kienzler et al. 2014). These datasets were merged and all building losses were indexed to the reference year 2006. Then the building loss ratio, i.e. the relative damage of each case, was derived by dividing the indexed building loss by the respective building asset value, which was estimated based on guidelines of the insurance sector (for details see Thieken et al. 2005 or Elmer et al. 2010) as replacement costs, and finally indexed to the year 2006.

The data were used to derive loss functions in two different ways. First, all available data with the required information, i.e. building loss ratio, water depth, contamination and private precaution, were considered. Secondly, only flood affected households which were located in the federal state of Bavaria were extracted, as Bavaria is very close to the study area in Tyrol and was supposed to be similar in regard to building and damage characteristics. In the first subset for all of Germany, 1121 cases were considered, while the Bavarian data subset was reduced to 415 cases (Cammerer et al. 2013a). Following the approach of Kreibich and Thieken (2008), a linear, a square root as well as a polynomial function were fitted to the data; each of them in two variants, i.e. accounting for contamination of floodwater or not. Additionally, the flood loss estimation model (FLEMO) for the residential sector that can also account for damage reduction or enhancement by private precautionary measures and contamination of the floodwater (see Thieken et al. 2008) was adapted for applications in Austria as outlined in Cammerer et al. (2013a). That model version is further referred to as FLEMO_AT_ or FLEMO_AT+_ in case effects of contamination and precaution are considered. Furthermore, three typically used depth-damage functions for the residential sector in Germany developed by MURL (2000),; ICPR (2001) and Hydrotec (2002), respectively, were applied.

Altogether, 57 variants of loss models were investigated on their fitness for correctly estimating the flood damage of the 2005 event in the study area:

- the three functions developed by MURL (2000), ICPR (2001) and Hydrotec (2002);
- four variants of FLEMO_AT_, i.e. with and without consideration of contamination and private precaution, each derived from two datasets (the German and the Bavarian dataset);
- four variants of the linear depth-damage function, i.e. with and without consideration of contamination, each derived from two datasets (the German and the Bavarian dataset);
- four analogue variants of the root function as well as of the polynomial function.

Each of these 19 models was combined with three specific building asset values (min, max and mean, see previous section). In this study, only loss models that were successfully validated for the flood event in 2005 as described by Cammerer et al. (2013a) were used to estimate present and future flood losses.

#### Risk calculation

Flood risk was quantified based on inundated areas and loss estimates of the 30-, 100-, 200- and 300-year flood of the current (as at 2006) and future (as at 2030) situation. In order to quantify risk and to compare changes in risk, risk curves were created showing the total direct residential building loss in the study area against its exceeding probability (i.e. return periods of the respective discharges at the gauge Lechaschau). In addition, the expected annual damage (EAD) was calculated as given in Merz and Thieken (2004).

### Flood scenarios and adaptation options

**Fig. 3:**
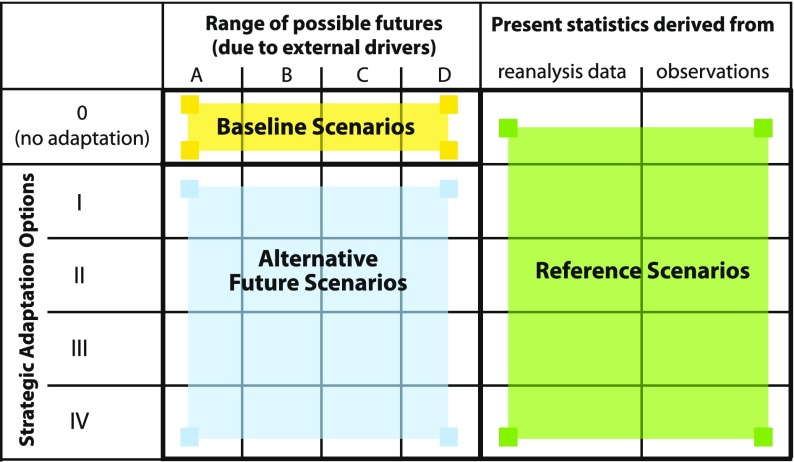
Conceptual framework for a scenario analysis on changing flood risks and adaptation options (modified after Luther and Schanze 2009)

The set-up of a scenario analysis currently differs in the literature, especially when adaptation options are considered. In this study, we are using the conceptual framework introduced by Luther and Schanze (2009), which is illustrated in Fig. 3. In this framework, reference, baseline and adaptation scenarios are distinguished. While reference scenarios describe the current flood risk based on historical data, observations or reanalyses, baseline scenarios quantify the general future flood risk in a projection period. In the baseline scenarios different, but reasonable futures are considered on the basis of consistent and combined storylines for different external drivers such as climate change and land use demand. The baseline scenarios should be selected with regard to the region under study and could be selected within a participatory framework.

In a further step, adaptation comes into play. While the baseline scenarios show the overall change due to external drivers, adaptation scenarios further estimate the effects of different adaptation options on the selected baseline scenarios. Options that are controllable at regional or local level should be chosen, e.g. land use policies, improved private precaution or strengthened technical protection measures. In our study, we solely look at the effects of non-structural measures.

Hence, our scenario analysis consisted of four steps. In the first step, the reference scenario in 2006 was calculated, i.e. flood risk was quantified with the successfully validated models and based on the 30-, 100-, 200- and 300-year flood discharges and resulting inundation areas considering structural protection measures and land use with building restriction policies as at 2006. The level of private precautionary measures and contamination of the floodwater was derived from a Tyrolean survey that was undertaken after the flood in 2005 (Schwarze et al. 2011).

In a second step, future flood risk was assessed for the year 2030. Annual maximum discharges from 2016 to 2045 were considered as being representative. In this step, a wide range of possibilities for all external drivers, i.e. climate change, land use change as well as economic development, was considered in the flood impact analysis. In detail, flood risk in 2030 was estimated considering structural protection measures as at 2006 and two different climate change scenarios based on the EH5OM modelling results with the A2-scenario and on HadGEM2 simulation runs with the A1B-scenario, respectively. The derived hydraulic scenarios were further intersected with four different land use scenarios for 2030 (see Model chain). Thereby, the assigned asset values for residential areas in the future, i.e. in 2030, were based on constant values, i.e. asset values at constant prices of reference year 2006, as well as on adjusted values, i.e. asset values adjusted by an increased GDP at prices of the reference year 2006. Furthermore, mean as well as minimum and maximum specific asset values were considered in order to account for uncertainty in the asset estimation as shown by Cammerer and Thieken (2013). The estimation of flood losses further combined these exposed asset values with the water depths of the hydrodynamic simulations by means of several loss models that were successfully validated for the flood event in 2005 (see Cammerer et al. 2013a and Calibration and validation of the model chain). Finally, the contribution of each driver to the overall flood risk was calculated.

The third step of the analysis aimed at defining a baseline scenario that represents a reasonable and consistent development path in terms of emission scenario, i.e. climate change, land use scenario and economic development for the study region. No adaptation measures were considered. In this paper, we investigated two baseline scenarios: In the first baseline scenario the modified 30-, 100-, 200- and 300-year flood according to the mean simulation with the model chain ‘HadGEM2 with A1B-emission scenario – EDS – HQsim – GEV’ assuming structural protection measures as at 2006 was combined with the most intensive urbanisation scenario ‘Overall Growth’ and current building restrictions. For the economic development constant values were assumed and the level of private precautionary measures and contamination were roughly taken as found in the Tyrolean survey after the flood 2005 (see Table 1). This baseline scenario represents a situation of rapid economic growth and globalisation of production and knowledge transfer processes, such as technology uptake that also includes the study area.

**Table 1:** Assumptions on shares of households that implemented private precautionary measures and/or suffered from oil contamination in the floodwater; see Data and Methods for explanations of scenarios

Share of households with specific characteristics	Reference scenario	Baseline scenario	Adaptation scenario ‘Resilience’	Adaptation scenario ‘Ignorance’ (Lost risk perception)
**In depth-damage functions that account for contamination**				
No contamination	72 %	70 %	90 %	50 %
With contamination	28 %	30 %	10 %	50 %
**In the loss model FLEMO**_**AT+**_				
No contamination, no private precaution	50 %	50 %	20 %	40 %
With contamination, no private precaution	21.5 %	20 %	10 %	40 %
No contamination, good private precaution	21.5 %	20 %	70 %	10 %
With contamination, good private precaution	8 %	10 %	0 %	10 %

In the second baseline scenario the modified 30-, 100-, 200- and 300-year flood according to the mean simulation with the model chain ‘EH5OM with A2-emission scenario – EDS – HQsim – GEV‘ assuming structural protection measures as at 2006 was combined with the slightly lower urbanisation scenario overall competition. Assumptions for building restrictions, economic development, private precautionary measures and oil contamination were the same as in the first baseline scenario. This second scenario assumes that the study region focuses more on local values, structures and traditions.

In the fourth and final step, the effect of different non-structural adaptation options on the two baseline scenarios was investigated. While the assumptions for climate change, land use change and economic development were not modified, adaptation was included by presuming different regulations for building restrictions as well as varying levels of private precaution. In total, two adaptation options were considered:

The first option represents a resilient society. In this option good risk awareness, e.g. due to frequent flooding and/or risk communication campaigns, is assumed which leads to a high level of private precaution that also prevents contamination of the floodwater by oil or other substances. This adaptation was implemented in the model chain by including improved building restrictions in the flood prone areas in 2030, i.e. settlements were forbidden in the red and yellow zones of the hazard zone maps. In addition, private precaution was assumed to increase considerably up to 70 %, while oil contamination of the floodwater rarely takes place, i.e. in only 10 % of the cases (see Table 1). The assumed values were taken from a survey in the catchments of the river Rhine and Moselle (in Germany; data subset of 2011 in Kienzler et al. 2014). This area is commonly regarded as being well-adapted to flood risk (e.g. Bubeck et al. 2013).

The second adaptation option represents ignorance or a lost risk perception, i.e. low risk awareness leads to a low level of private precaution, but a high percentage of contaminated floodwater. Structural protections as well as building restrictions remained as at 2006. In detail, it was assumed that only 20 % of the affected households undertake private precautionary measures and that at 50 % of the affected buildings the floodwater was additionally contaminated by oil (see Table 1). Similar values were found during a severe flood event in 2002 in regions, where flooding had not been experienced for a long time (see Thieken et al. 2007).

Since only the loss model FLEMO_AT+_ is capable of considering effects of contamination and precaution on the flood loss, the adaptation options were calculated with the variant of this model that performed best during the model validation.

## Results

The results of the case study are presented in six sections. The first section summarises the calibration and validation of the model chain. The second section illustrates the current flood risk, while in the next three sections the contributions of different external drivers – climate change, land use change and economic development – on the changes in flood risk of the study area are presented. The sixth section finally focuses on the effects of non-structural adaptation options on the flood risk.

### Calibration and validation of the model chain

As outlined in Data and methods, the Expanded Downscaling (EDS) model was calibrated and validated by ECMWF-interim data from 1989 to 2005. Table 2 provides performance statistics, i.e. correlation coefficients of observed and simulated areal precipitation, which is defined as the daily mean of all precipitation stations (Dobler et al. 2013). Since at least a decade of daily data is necessary to calibrate the EDS model, only five years could be used for validation, which is quite a short period when evaluating extreme events. Nevertheless, the observed precipitation is reproduced fairly well by the EDS model with reanalysis data. In the period from 1989 to 2005, the correlation coefficients of observed and simulated areal daily precipitation range from 0.76 to 0.78 (Table 2). Dobler et al. (2013) showed that there is also a good agreement between observed and simulated values in the highest percentiles.

**Table 2:** Performance statistics of the calibration and validation of the expanded downscaling model (EDS) and the hydrological model HQsim (ECMWF stands for European Centre for Medium-Range Weather Forecasts)

Model and input	Performance measure	Calibration (1989–2000)	Validation (2001–2005)	Total (1989–2005)
EDS driven by ECMWF-data	Correlation of observed and simulated areal daily precipitation	0.78	0.76	0.78
HQsim driven by observed station data	Nash-Sutcliffe-Coefficient of observed and simulated daily runoff	0.85	0.88	0.86
HQsim driven by downscaled ECMWF-data	Nash-Sutcliffe-Coefficient of observed and simulated daily runoff	–	–	0.73

In general, downscaling precipitation extremes is a challenging task in alpine areas and is subject to large uncertainties. Recently, several investigations have reported large model biases when focusing on precipitation extremes (e.g. Smiatek et al. 2009). The results of this investigation show that the EDS model performed very well in reproducing observed precipitation (extremes). The biases of all simulations are within an acceptable range, even for rare events, e.g. that one with a 20-year return period (see Dobler et al. 2013). Thus the downscaled meteorological data can serve as input data for the hydrological model.

The next element of the model chain, the hydrological model HQsim, was first analysed by a sensitivity analysis by Dobler and Pappenberger (2013). The results showed that parameters affecting snow melt and processes in the unsaturated soil zone were of high significance in the Upper Lech catchment. The parameter meltfunc_max, which defines the maximum degree-day factor, was found to be of particular importance. While parameters affecting snow melt showed clear temporal patterns in the sensitivity throughout the year, the importance of parameters affecting processes in the unsaturated soil zone did not vary in importance throughout the year (Dobler and Pappenberger 2013). These findings considerably helped to improve the model calibration that was performed on the basis of observed meteorological and hydrological data.

Generally, a good agreement between observed and simulated runoff data was obtained. The hydrological simulations revealed slight weaknesses in the simulation of flood peaks during winter. However, as these events are usually small to medium flood events in comparison to summer floods, they may not cause large damage. Hence, their influence on the estimation of flood risk is limited. The Nash-Sutcliffe efficiency (NSE) criterion, which was used to quantify the model performance, is 0.86 for the period from 1989 to 2005, indicating that the model performs well in this complex Alpine watershed (see Table 2).

Finally, the performance of the model chain consisting of the EDS and HQsim models was tested in reproducing observed runoff data. It can be seen that the modelling chain captured hydrological processes well with a NSE of 0.73 for the calibration and validation period (Table 2). The performance of the HQsim simulation driven with downscaled reanalysis data showed slight weaknesses when focusing on very extreme floods, such as those in 1999 and 2005, which had return periods of multiple centuries (Dobler et al. 2012). This is mainly due to the relatively short calibration and validation period used in this study.

Annual maximum discharges series (AMS) were derived from the simulated discharge time series and flood frequencies were analysed by fitting a GEV-distribution to the respective AMS (see Current flood risk). Peak flows with return periods of 30, 100, 200 and 300 years delivered finally the basis for the flood impact analysis, e.g. inundation modelling and damage estimation. These two elements of the model chain were validated separately for the severe flood event in August 2005.

For the hydraulic simulation of the flood event in 2005, the structural measures implemented at the time of the event were considered in the geometry of the hydraulic model. Two simulation runs were performed. In the first simulation, the levee failures that occurred in the community of Pflach in 2005 (Kröll 2007) were accounted for by artificially opening two breach locations. In the second simulation run, no dike breaches were included. The performance of the two runs was validated by comparing the simulated water depths with observed data at eleven water marks and by comparing the simulated flood extent with the real extent that was mapped for a part of the study area. Given this scarce data base for validation, Cammerer et al. (2013a) showed that both simulation runs performed similarly: The mean absolute error (MAE) of the water depths at the water marks amounted to 0.38 m and the root mean square error (RMSE), which emphasizes larger deviations, is 0.51 m in both runs. The flood area index could only be determined for a part of the study area and amounted to 84 %. This rather low value is due to the shortcomings of the mapping procedure outlined by Ebner et al. (2007). Hence, the error statistics indicate a reasonable fit of the hydraulic model.

As outlined in the section Data and Methods, a total number of 57 variants of loss models were tested by Cammerer et al. (2013a) for their ability to estimate the residential damage that occurred in 2005. Reliability was judged by a 95 % confidence interval that was created by a bootstrap exercise with the actual loss data recorded in the study area by the Austrian Disaster Fund.

Out of the three commonly applied depth-damage functions, only the loss function of ICPR (2001) lies within the 95 % confidence interval of the reported loss. The empirically derived depth-damage functions (linear, polynomial and square root) only reliably estimated the reported damage if the functions had been derived from the Bavarian sub-dataset, i.e. from damage data of a region that is similar to the study region with regard to flood and building characteristics. The same holds for the adapted multi-criteria flood loss estimation model FLEMO_AT(+)_. Altogether, the model validation revealed that 29 out of 57 damage model variants delivered reasonable loss estimates for the 2005 event (Cammerer et al. 2013a). The procedure illustrated the importance of the site-specific evaluation of flood loss models.

### Current flood risk

**Fig. 4:**
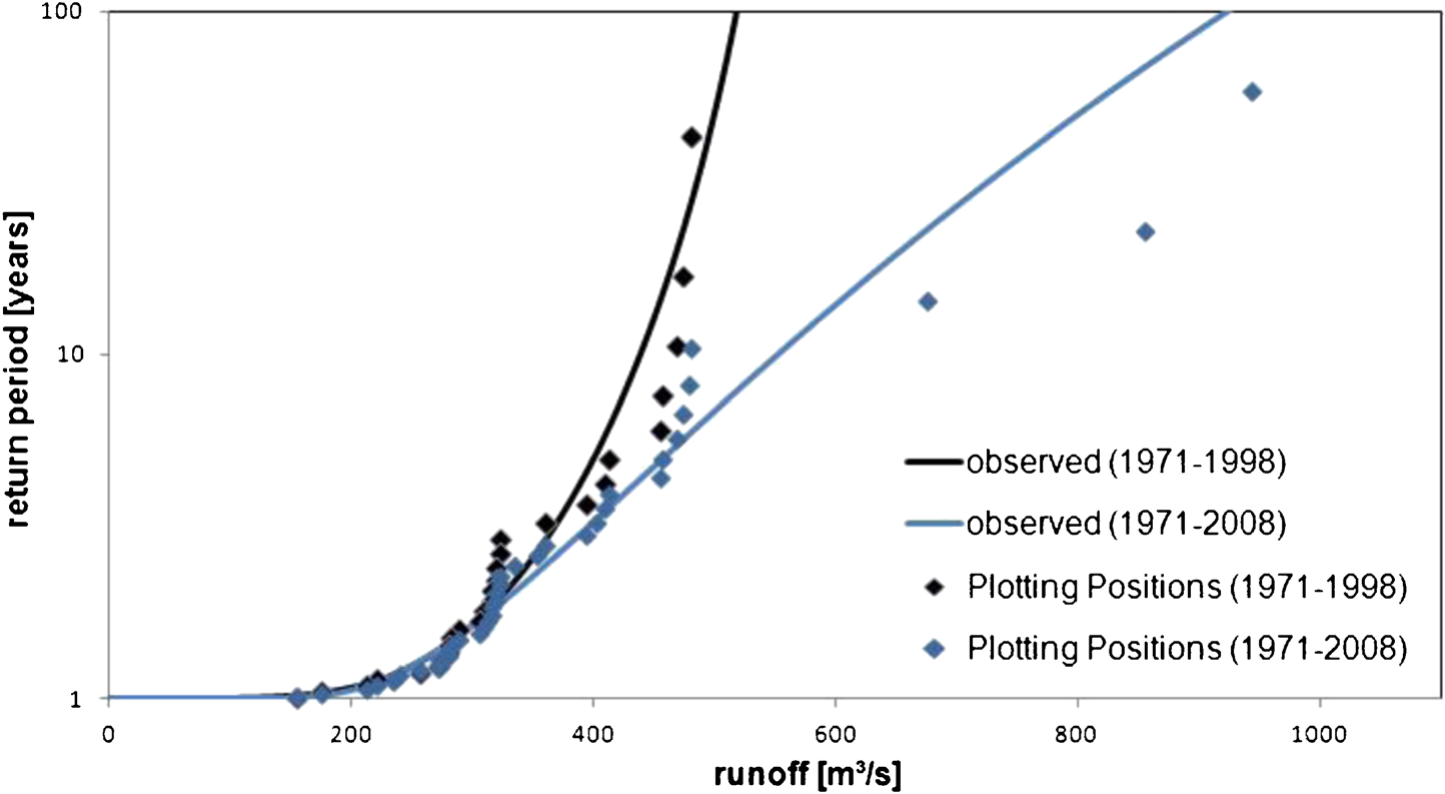
Flood frequency analysis for different time slices at the Lechaschau gauge (Source: Thieken et al. 2011)

To assess changes in flood hazards and risks, the current situation, i.e. the flood hazard and risk around the year 2006, has to be quantified first. For this, a GEV-distribution was fitted to the annual maximum discharge series of observed runoff data. When two different time slices of data from the Lechaschau gauge, 1971 to 1998 and 1971 to 2008, respectively, were used, a wide range of uncertainty was obtained as shown in Fig. 4. The occurrence of the three severe floods in 1999, 2002 and 2005 decisively changed the distribution. On the basis of data from 1971 to 1998, a return period of more than 1,000 years was assigned to the flood in 2005, while a return period of only 110 years is assigned to the same flood when data from 1971 to 2008 were considered. Therefore, historical data were included in the analysis. For this, runoff records downstream of Lechaschau/Reutte, i.e. from the Füssen gauge in Germany, were used. Here, data have been available since 1901; since 1954 measurements have, however, been influenced by backwater effects. Therefore, data were only used to identify strong historic flood events.

The time series at the Füssen gauge revealed that a flood with a similar intensity like the one in 1999 occurred in 1910. Since this historical event was also confirmed by Meier (2002) for the Upper Lech Valley, it was included in the flood frequency analysis at the Lechaschau gauge by using the procedure of DVWK (1999). With this approach a return period of about 330 years is assigned to the flood in 2005.

Further, it was assumed that the two curves shown in Fig. 4 can be regarded as an envelope of the real flood frequency distribution at the Lechaschau gauge, since the series of the time period 1971 to 1998 contains comparatively low flood discharges, while the series from 1971 to 2008 includes three severe flood events. Considering this, the inclusion of historical information significantly improves the estimation of events with higher return periods.

**Fig. 5:**
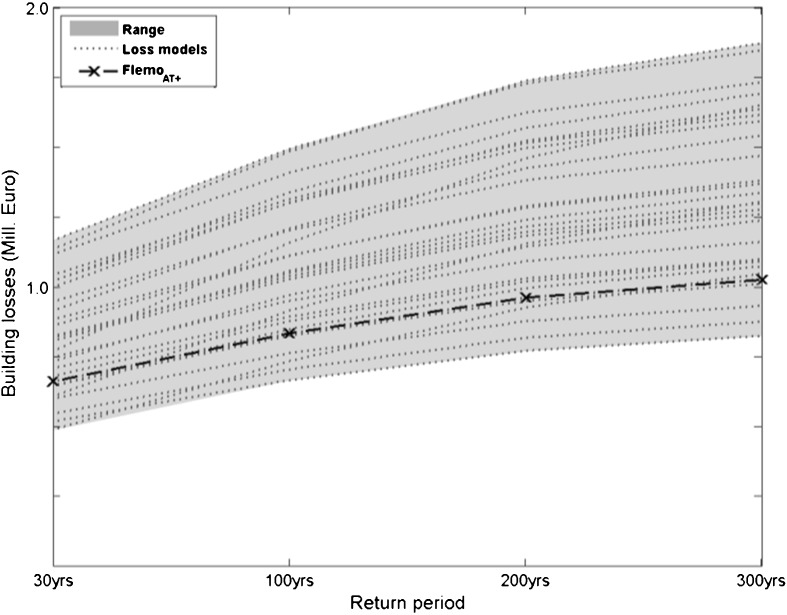
Current risk curve considering residential building losses in the study area with uncertainty bounds based on the range of 29 successfully validated loss model combinations. The model FLEMO_AT+_ is highlighted since it was further used to estimate effects of adaptation options on the flood risk

The 30-, 100-, 200- and 300-year peak discharges were extracted from the flood frequency analysis and were used as input for the hydrodynamic simulation that accounted for structural defence measures as implemented by 2006 (further referred to as reference scenario). The resulting inundation maps were further used to estimate building losses in the residential sector. In this step, only the successfully validated loss models were used. The resulting risk curves are depicted in Fig. 5.

### Changes in the flood hazard (climate impacts)

To simulate effects of climate change on the flood hazard, the calibrated and validated model chain GCM with emission scenario – EDS – HQsim was applied using control and simulation runs of the EH5OM model with the emission scenario A2 as well as HadGEM2 with the emission scenario A1B.

To assess changes in the flood frequencies, the GEV-distribution was fitted to the annual maximum discharge series of both, the control and the scenario simulations. Relative changes in the peak flows at certain return intervals for the control and scenario simulations were calculated. Table 3 shows the calculated changes. These values were used to alter the flood peak discharges derived from the series with historic flood records and served as input for the hydraulic modelling. It can be seen that impacts of climate change on flood frequencies in the near future is low. A clear tendency of peak discharges cannot be derived from the simulations. Whether this will change in the farther future, needs further investigation.

**Table 3:** Relative changes in peak flows of the control and scenario simulations (from 2016 to 2045) at certain return periods, based on the downscaled outputs of the general circulation models EH5OM (with emission scenario A2) and HadGEM2 (with emission scenario A1B), hydrological modelling with HQsim and the generalised extreme value (GEV) distribution; see Data and Methods for explanations of abbreviations and models

Return period of peak flow [years]	EH5OM (A2)	HadGEM2 (A1B)
30	−3.9 %	−8.4 %
100	−0.2 %	−8.0 %
200	+3.4 %	−7.6 %
300	+5.9 %	−7.6 %

### Changes in land use and exposed asset values

**Fig. 6:**
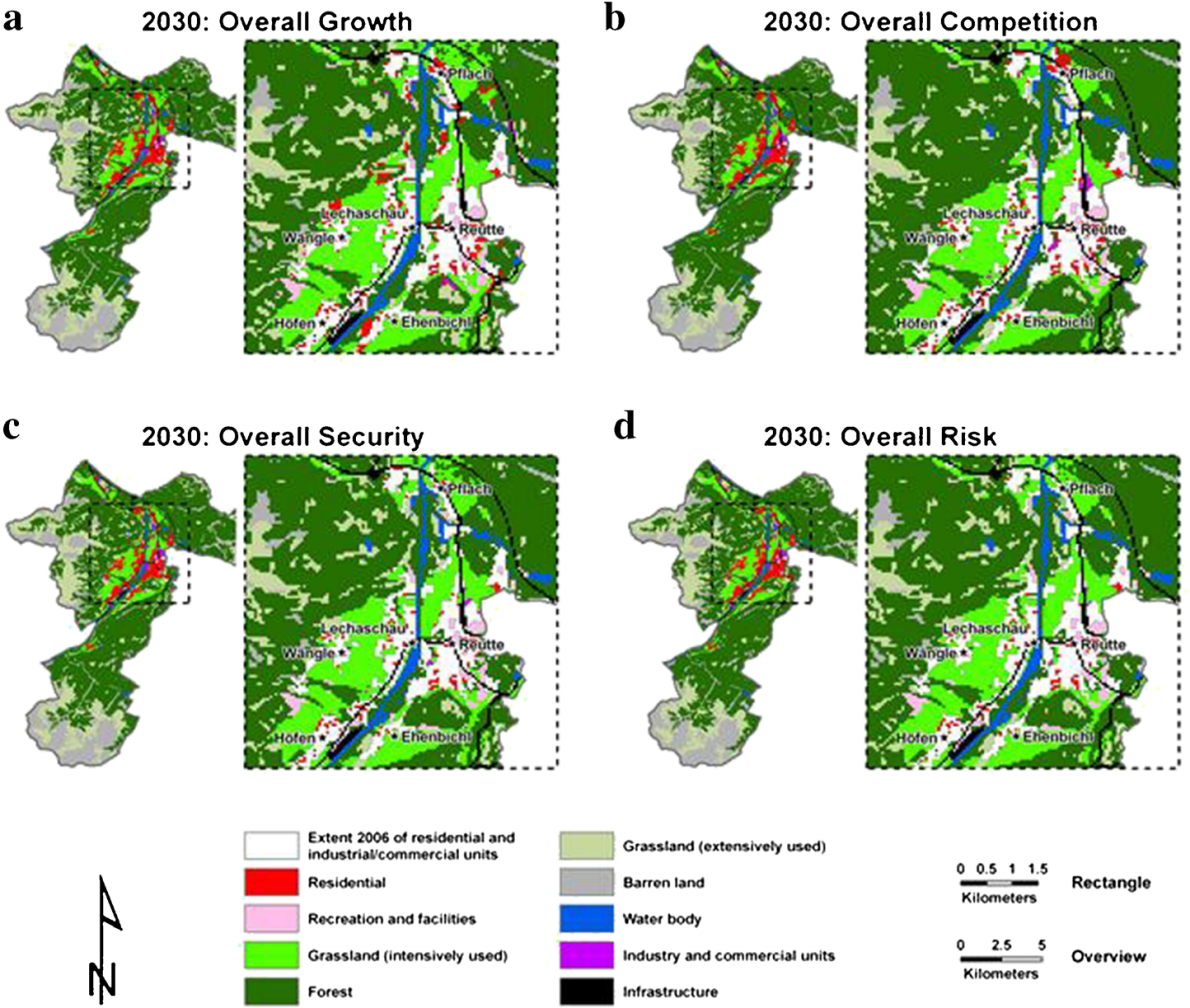
Land use scenarios in 2030 for the area of Reutte with rectangle of the settlement area based on current spatial policy and the storylines **a** Overall Growth, **b** Overall Competition, **c** Overall Security and **d** Overall Risk; note that the extent of residential and industrial/commercial units in 2006 are displayed in *white colour*

For the future scenario generation, the land use demand of the different land use types had to be assessed. Considering four different national projections of the Austrian Conference on Spatial Planning (ÖROK) and the current spatial policy in simulations with DynaCLUE (see Data and methods), spatially explicit land use changes by 2030 were derived for the study area as depicted in Fig. 6. The maps reveal a wide range of potential land use changes. Although the simulated land use changes between 2007 and 2030 are not as intensive as in the historic time span of 1971 to 2006 (see Cammerer and Thieken 2013), they are still notable – depending on the assumed projection of land demands. Concerning urbanisation, the historical annual growth rate of residential areas, for instance, is not more than one and a half times higher than in the strongest urbanisation scenario ‘Overall Growth’. However, in comparison with the weakest urbanisation scenario ‘Overall Risk’, the factor amounts to 5.7. In case of industrial and commercial units, the observed mean annual growth rate is even more intense than for residential areas.

**Fig. 7:**
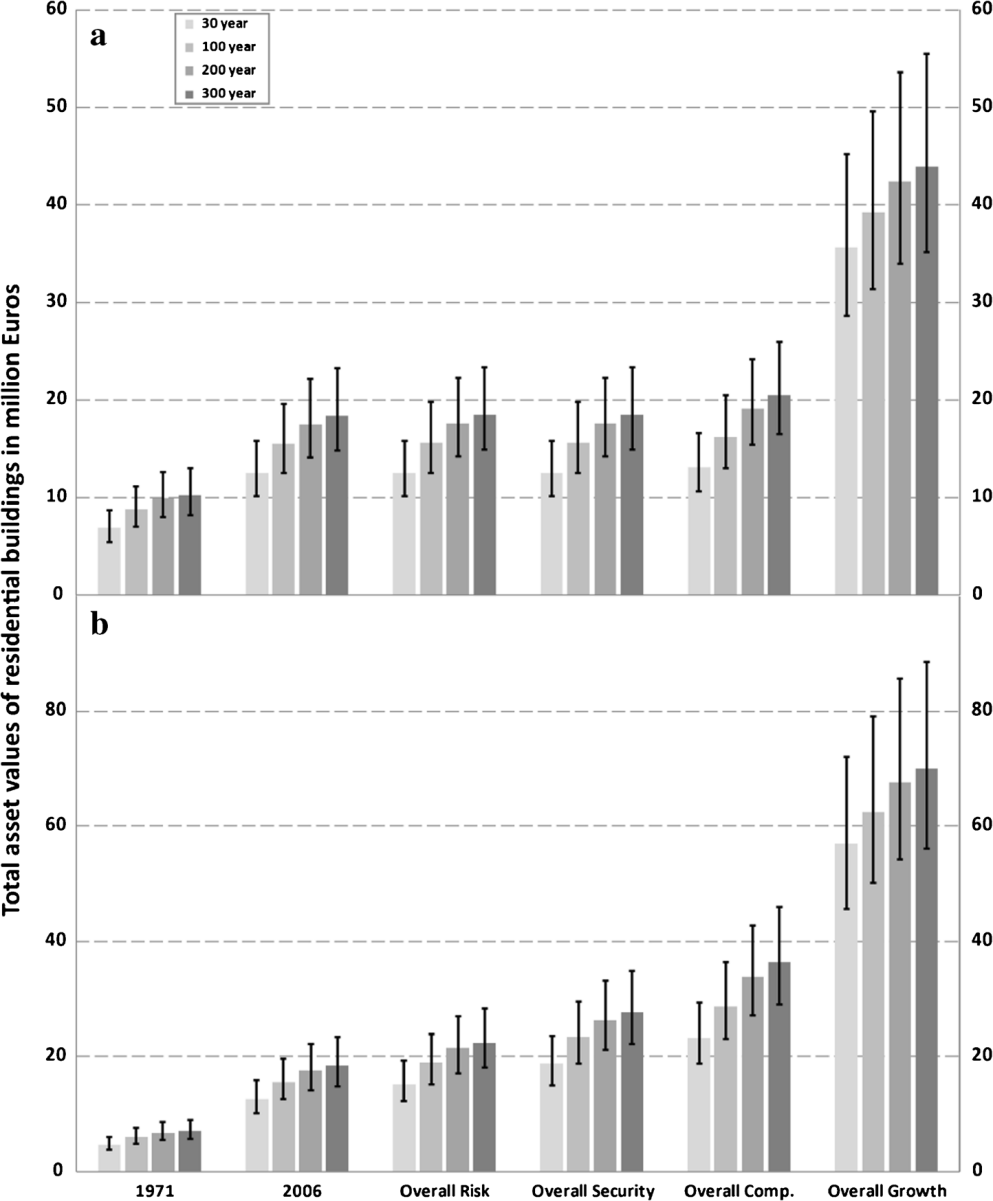
Total asset values with range (by using minimum and maximum unit values) for residential buildings in million Euros and constant values **a** as well as adjusted values **b** for different points in time within the inundated area of the 30-, 100-, 200- and 300-year flood of the reference period (i.e. around 2006)

To filter out the effect of the development of the asset values at risk from potential changes in the flood hazard, only the inundation scenarios for the current situation, i.e. without any impacts of climate change, were combined with the land use scenarios. The results of the historic and potential future development in the assets at risk are shown exemplarily by the total building values in the four inundation scenarios for different points in time (Fig. 7). When the building values at risk are compared with the replacement values of 2006 (constant values) considerable changes in the historic time span (1971 to 2006) can be detected (Fig. 7a). In this period of 35 years, an annual growth rate of more than  ~ 2.2 % was observed in all four inundation scenarios.

In the simulated period (2007 to 2030), the asset value development depends very much on the underlying land use scenario. In the two rather moderate land use scenarios ‘Overall Risk’ and ‘Overall Security’, the flood-exposed building values are almost constant until 2030 in comparison to 2006. In the scenario overall competition, however, the building values grow slightly in the potential inundation areas (0.2 to 0.4 % per year) between 2007 and 2030. Only in the most extreme urbanisation scenario ‘Overall Growth’, the annual growth rate jumps up remarkably, which is even higher (i.e. 5.8 to 7.7 %) than in the historical period (Fig. 7a).

When the residential building values at risk are corrected by means of the real GDP (adjusted values), both for 1971 and for the four scenarios for 2030, the relative changes in the flood exposed building values are considerably larger due to the consideration of the economic development in this period (Fig. 7b). Thus the historical changes between 1971 and 2006 already account for an increase of ~ 4.5 % per year in the areas at risk. Between 2007 and 2030 the range of the annual growth rate of the building values at risk is estimated to be between ~ 1 % (Overall Risk) and ~ 2 % (Overall Security) in the two moderate land use scenarios. For the scenario ‘Overall Competition’, an annual increase of 3.5–4.1 % of the building values was derived. In the strongest land use and economic growth scenario ‘Overall Growth’, the annual increase even amounted to 11.7 % and 14.8 % (Fig. 7b). However, the application of a minimum or maximum unit asset value instead of the average value (see ranges in Fig. 7) shows that the total building values at risk may be subjected to considerable uncertainties.

The investigation of the asset values at risk reveals a remarkable increase in residential building values within the present flood zones. For the future, a further growth of asset values in the flood-prone areas can only be detected for the strongest land use scenarios. However, an additional consideration of economic growth by adjusted values leads to a further and clear rise in the assets at risk, particularly in the projected time span (2007 to 2030) and the strongest urbanisation and economic growth scenarios.

### Changes in flood risk – sensitivity analysis of external factors/drivers on changes in flood risk

**Fig. 8:**
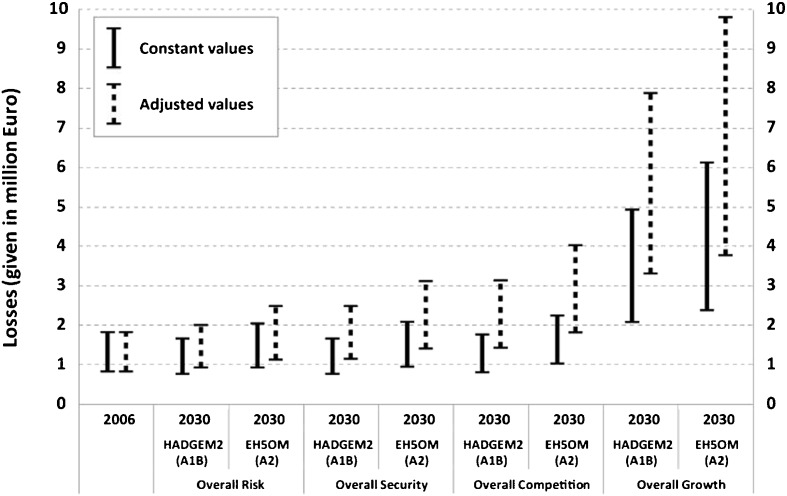
Estimated flood losses for residential buildings based on mean constant values and adjusted values (with range by using minimum, mean and maximum specific values and the loss models/functions that were successfully validated for the 2005 event) for a 300-year flood in 2006 and in 2030 considering two different general circulation models as well as four land use scenarios. Note that EH5OM (A2) combined with ‘Overall Competition’ as well as HADGEM2 (A1B) combined with ‘Overall Growth’ were further used as baseline scenarios (see also Data and Methods)

Due to the variety of scenarios that were considered in each step of the model chain, a variety of damage estimates was obtained for each return interval. This is exemplarily shown for the most intense flood event, i.e. the 300-year flood, in Fig. 8. Thereby, the residential building values were based on constant values as well as on adjusted values and also included the range of the specific asset values. Building damage was estimated by means of all loss models/functions that were successfully validated for the 2005 event.

It can be seen from Fig. 8 and Table 4 that potential damage on residential buildings grows only slightly between 2006 and 2030, when constant values were assumed – except for the strong urbanisation scenario ‘Overall Growth’. Furthermore, loss estimates based on the climate scenario HADGEM2 (A1B) were generally lower than estimates based on EH5OM (A2), which is consistent with the data presented in Table 3.

**Table 4:** Relative differences (in %) of potential flood losses in residential areas between 2006 and 2030 for four recurrence intervals depending on different land use and climate scenarios (see Data and methods for explanations of abbreviations) as well as on the 29 successfully validated loss models/functions with constant values (left) or adjusted values (right, in parenthesis)

Scenarios		Relative differences (in %) of potential residential flood losses between 2006 and 2030							
Land use	Climate	30-year event		100-year event		200-year event		300-year event	
Overall risk	HADGEM2 (A1B)	−13 to −7	(6 to 13)	−9 to −8	(10 to 11)	−11	(7 to 8)	−9 to −8	(10 to 11)
	EH5OM (A2)	−5 to −3	(15 to 17)	0	(21 to 22)	4 to 5	(26)	12	(35 to 36)
Overall security	HADGEM2 (A1B)	−13 to −7	(30 to 39)	−9 to −8	(36 to 37)	−11	(32 to 33)	−9 to −8	(35 to 37)
	EH5OM (A2)	−5 to −3	(41 to 44)	0	(50)	4 to 5	(55 to 56)	13 to 14	(69 to 71)
Overall compet.	HADGEM2 (A1B)	−9 to −2	(60 to 73)	−5 to −4	(68 to 69)	−7 to −6	(65 to 67)	−4 to −2	(70 to 73)
	EH5OM (A2)	−2 to 1	(74 to 79)	4	(84)	13	(99 to 100)	23 to 24	(117 to 120)
Overall growth	HADGEM2 (A1B)	211 to 219	(396 to 408)	171 to 180	(332 to 347)	153 to 164	(304 to 321)	150 to 166	(299 to 324)
	EH5OM (A2)	219 to 228	(408 to 423)	185 to 199	(355 to 376)	181 to 212	(348 to 398)	187 to 230	(357 to 426)

For example, potential losses of the land use scenario ‘Overall Competition’ were 23–24 % higher (EH5OM with A2) in comparison to the potential losses in 2006 than the estimates from the HADGEM2 (A1B) simulation run (−4 to −2 %; Table 4). In the strongest urbanisation scenario, however, the differences of both climate scenarios to 2006 were considerable, amounting to 187–230 % as well as 150–166 %, respectively (Table 4).

When all loss estimates were based on adjusted values, the increase between 2006 and 2030 was stronger due to the consideration of the economic development (Fig. 8 and Table 4). Then potential losses of the weakest urbanisation scenario ‘Overall Risk’ were higher for both climate scenarios than in 2006. The loss estimates resulted in 35 to 36 % (HADGEM2 with A2) or 10 to 11 % (EH5OM with A1B) higher potential damage to buildings than in 2006 (Table 5). For the most intense urbanisation scenario ‘Overall Growth’, the estimated potential loss even tripled for both climate scenarios (Table 4).

**Table 5:** Estimates of expected annual damage (EAD) of the baseline scenarios and two adaptation options as explained in Data and Methods. The EAD values were calculated with estimates of the flood loss model FLEMO_AT+_ assuming minimum asset values. EAD estimates were rounded to thousand Euros and are given in prices as at 2006. Percentages refer to the baseline scenario as at 2006

		Range of possible futures (due to external drivers)		Past and present risk derived from observed data	
		EH5OM (A2), Overall competition	HADGEM2 (A1B), Overall growth	Land use as at 2006	Land use as at 1971
No adaptation	Baseline	25 000 € (104 %)	70 000 € (292 %)	24 000 € (100 %)	13 000 € (54 %)
Strategic adaptation options	Resilience	18 000 € (75 %)	49 000 € (204 %)	17 000 € (71 %)	9 000 € (38 %)
	Ignorance (Lost risk perception)	29 000 € (121 %)	82 000 € (342 %)	28 000 € (117 %)	15 000 € (63 %)

Nevertheless, it is apparent that all damage estimates were associated with a relatively high uncertainty, resulting from the range of specific asset values, the different loss functions as well as the climate and land use scenarios. Thus the variation of absolute damage estimates was especially high when the strongest urbanisation and economic development scenario was taken into account.

Figure 8 and Table 4 indicate that especially the effects of land use change and economic development are responsible for the potential increase in losses to residential buildings in the future. While the application of two different climate models and emission scenarios (HADGEM2 with A1B and EH5OM with A2) resulted in rather low differences of the loss estimates, the use of different land use scenarios in combination with associated economic development had a tremendous effect on these estimations.

**Fig. 9:**
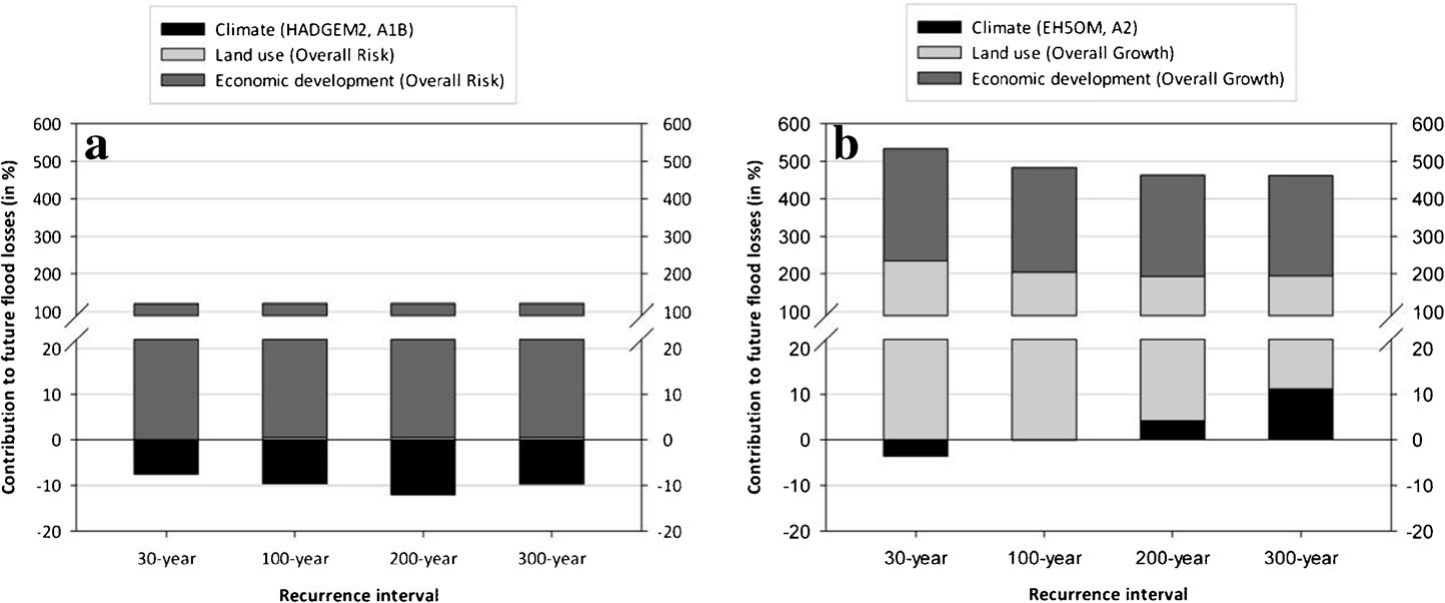
Relative contribution of the single effects of changes in climate, land use and economic development on future flood risk based on very conservative assumptions (**a**) and more extreme scenarios (**b**); see Data and Methods for explanations of abbreviations and model assumptions

In order to separate the contribution of the single effects, i.e. climate change, land use change and economic development, on the future flood risk, we combined the most contrasting land use scenarios (overall risk and overall growth) with the present flood hazard to isolate the full range of potential effects from land use changes on flood risk. On the one hand, this was done by means of constant values to isolate effects of land use change. On the other hand, these calculations were performed assuming adjusted values to separate the effect of economic development. For the estimation of the contribution of climate change, in contrast, we combined the outcome of the two climate models (HADGEM2 with A1B and EH5OM with A2) with the land use pattern as at 2006. Thus we derived the potential range of changes in flood risk with the most conservative scenarios (Fig. 9a) and with the most extreme scenarios (Fig. 9b). In Fig. 9, however, the effect of the range of asset estimates and flood loss functions was neglected. All loss calculations were carried out assuming the mean specific asset values and applying the polynomial function that also accounts for contamination of the floodwater. This model was chosen since it delivered the best estimate for the damage of the event in 2005.

As illustrated in Fig. 9, the isolated impact of climate change by 2030 on flood risk is comparatively small (Fig. 9b) or even negative (Fig. 9a). By 2030, impacts of climate change on flooding may be masked by the large uncertainty of climate simulation. In contrast, land use change can contribute considerably to future flood risk, when the strongest urbanisation scenario is assumed (Fig. 9b). In combination with the economic development, which has a nearly similar relative effect, the cumulative impact on flood risk may be remarkable, e.g. an increase of 484 % in case of a 100-year flood compared to 2006. However, in case of the conservative land use scenario ‘Overall Risk’ only the effect of economic development on the underlying asset values sticks out, while the contribution of land use is almost negligible and the influence of climate change is even negative (Fig. 9a).

The analysis reveals that in the near future changes in flood risks are governed by economic development and land use changes. Since these parts of the model chain could not be validated, further research on model performance and sensitivity is needed.

### Adapting to a changing flood risk

For the analysis of adaptation options, baseline scenarios that represent a consistent and reasonable development path in the region under study were chosen first. In this study, HADGEM2 with the A1B-scenario combined with the land use scenario ‘Overall Growth’ as well as EH5OM with the A2-scneario combined with ‘Overall Competition’ (see Flood scenarios and adaptation options) were selected. While the first baseline scenario assumes a continuous growth on both, the global and the regional scale with an equalisation of regions, the second scenario assumes a stronger focus on regional traditions and values. Hence, pressure on land is high in growing regions, whereas other regions are faced to migration and shrinking phenomena.

**Fig. 10:**
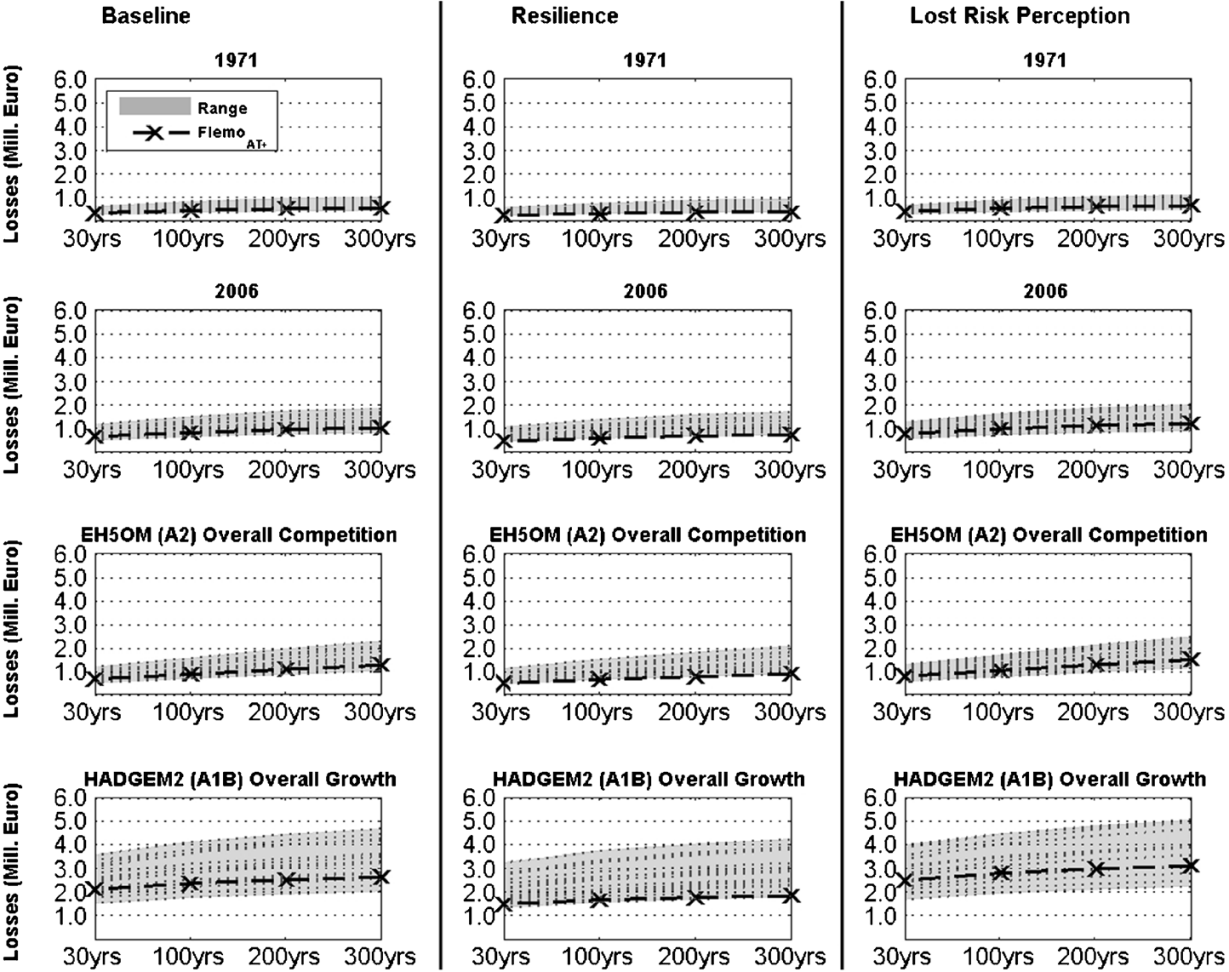
Loss estimates for flood events of different return periods and for the two baseline scenarios as well as for the two adaptation options that are explained in Data and Methods. Estimates with the flood loss estimation model FLEMO_AT+_ and minimum asset values are highlighted since this variant was used for the calculations of the expected annual damage presented in Table 5

As outlined in Flood scenarios and adaptation options, the effects of two adaptation options, ‘Resilience’ as well as lost risk perception, reflecting increased precaution and ignorance, respectively, were studied on both baseline scenarios. The results are presented as risk curves (Fig. 10) and as expected annual damages (EAD; Table 5). The loss estimation was performed with the 29 models/functions that were successfully validated for the 2005 event. Effects of contamination of the floodwater and private precaution (see Table 1) were considered where possible, i.e. in FLEMO_AT+_ as well as in depth-damage functions that also account for contamination of the floodwater. Since the effect of both, contamination and private precaution, could only be considered by the loss model FLEMO_AT+_, the outcomes of this model are highlighted in Fig. 10. The EAD calculations in Table 5 are also based on this model. In this model variant, minimum asset values were assumed, since this variant better estimated the damage of the 2005 event than other model variants.

As illustrated in Fig. 8 and Table 4, the first baseline scenario (HADGEM2 with A1B and Overall Growth) resulted in a considerable increase in potential flood losses (in constant values and prices for the reference year 2006), while the second baseline scenario (EH5OM with A2 and Overall Competition) only led to a slight increase. In comparison to the flood risk in 2006, this amounts to 292 % and 104 % of the flood risk in 2006, respectively (Table 5). It has to be acknowledged that flood risk had already almost doubled between 1971 and 2006 due to land use development (Table 5).

It is clearly illustrated in Fig. 10 that flood damage could be considerably reduced by non-structural measures such as stronger building restrictions in flood prone areas as well as a high level of risk awareness and private precaution. This holds for all baseline scenarios as well as in comparison to the option ‘Lost risk perception’. With regard to the expected annual damage, the resilience option reduced the EAD of all baseline scenarios by around 30 %. In contrast, the ignorance option (lost risk perception) increased the EAD by 17 % (see Table 5).

This analysis illustrates that non-structural measures that strengthen private precaution can reduce future flood risk regardless of climate or land use changes and can therefore be regarded as so-called no-regret measures, while ignoring flood risk always leads to increasing losses.

## Discussion

Data and knowledge on climate change impacts on flood risks as well as on the costs of appropriate adaptation are currently not transparently available (Hall et al. 2012). It was the aim of this study to partly fill this gap by using the Tyrol Lech catchment as an example. As risk includes aspects of flood hazard as well as vulnerability, the modelling approach within this study was tripartite: first, it was aimed at examining the impacts of climate change on the frequency-magnitude relationship of floods. Secondly, shifts in damage potential due to land use changes and economic development were to be investigated and quantified. Finally, it was aimed at quantifying future flood risks. Above all, effects of non-structural adaptation measures on the flood risk were investigated.

To reliably assess future flood risk, complex model chains have to be applied, including the linkage of global circulation modelling results with downscaling methods and hydrological models as well as frequency analysis, hydraulic modelling and damage estimation. Furthermore, land use changes, associated socio-economic developments and asset values have to be considered in damage and risk estimation. The model chain that is illustrated in Fig. 2 was successfully established and applied to the study area, although some drawbacks of the used methods and limitations of the results still remain.

To begin with, downscaling precipitation extremes is a challenging task and subject to large uncertainty, especially in mountainous areas. The results of our investigation showed that the EDS model performed well in reproducing observed precipitation, although the calibration period of the downscaling model was rather short (i.e. from 1989 to 2000). In summary, EDS is a valuable tool for studying the effects of climate change on precipitation extremes the local scale even in mountainous areas. Model calibration and validation could be further improved by longer periods of reanalysis data from ECMWF.

The downscaled precipitation and temperature time series were further used as input data for the conceptual semi-distributed rainfall-runoff model HQsim. Before running the model with these input data, investigations with different sensitivity analysis techniques were found to improve the understanding of the HQsim model and helped to accelerate its calibration and consecutive applications. In general, a good agreement between observed and simulated runoff data was obtained. However, the performance of the HQsim simulations driven with downscaled reanalysis data showed slight weaknesses when focusing on very extreme floods, such as those that occurred in 1999 and 2005. This is mainly due to the relatively short calibration and validation period used in this study, which implies that the model behaviour does not cover the totally possible natural variability and thus creates considerable uncertainty for events with high return periods. Again, longer periods of high-quality reanalysis data such as ERA-interim data would help to overcome this problem.

Discharge time series were then used to derive a flood frequency distribution. For this, the GEV-distribution was applied to annual maximum discharge series that were derived from observed discharges at the Lechaschau gauge. Since the occurrence of several severe floods between 1999 and 2005 decisively changed the distribution, data on historic flood events were included to get a more robust frequency distribution. Although this approach considerably helped to improve flood frequency estimations, questions concerning the stationarity of discharge data are still open: the benefits of enlarging the time series may result in the drawback that the time series may not be representative for the present conditions (Merz and Thieken 2009).

Different approaches how to include climate change in flood frequency distributions can be found in the literature (compare e.g. te Linde et al. 2011; Elmer et al. 2012). Since discharge time series that result from the model chain GCM (EH5OM with A2, HadGEM2 with A1B) – Downscaling (EDS) – hydrological model (HQsim) must not be seamlessly combined with observed discharge series, the relative changes in the peak flows with certain exceedance probabilities in the control (1971–2000) and the scenario (2016–2045) simulations were calculated in this study (see Table 3). These changes were then added to the peak flows that had been derived for the same exceedance probabilities from the observed data (1971–2008, including historic events). By this approach altered flood frequency-intensity information for the near future (representing the year 2030) could be reliably derived and further used in the hydraulic simulation.

In order to transform peak discharges into inundation areas and depths, the two-dimensional model Hydro_AS-2D was implemented in the region of Reutte, which is the most important settlement area in the study region. The hydrodynamic model was capable of simulating the most recent flood event in 2005 with acceptable quality, although data on cross-sections and structural protection measures were incomplete and hampered the model application. Therefore, effects of adaptation by structural protection measures could not be investigated in detail. Nevertheless, various flood scenarios were simulated for different recurrence intervals (30-, 100-, 200-, 300-year flood) for the present, i.e. in 2006, as well as for the future situation, i.e. in 2030, considering two different climate change scenarios and the most recent structural protection measures. With this step, the flood hazard analysis was completed. Inundation scenarios could further be used for exposure and damage analysis.

In order to not only account for impacts of climate change, considerable efforts were made in this study to simulate future land use changes. For this, the land use model Dyna-CLUE was applied to the region of Reutte including current spatial policies, e.g. area zoning plans and hazard zones, and taking four different national projections of land demand from ÖROK (2008) as basis for the spatially explicit scenario development. With this approach, explicit land use simulations with a spatial resolution of 50 m were conducted by 2030. Due to limitations of assumptions and data, it was not possible (nor reasonable) to simulate land use change beyond 2030 at this local scale – in contrast to the flood hazard analysis that could – in principle – be performed by 2100.

Another difficulty is the missing validation of the land use model due to the lack of further land use data sets and other input data. Although the applied land use model was validated successfully in many case studies worldwide (e.g. Pontius et al. 2008), the validation is, of course, site-specific as the model can behave differently in other settings (Pontius et al. 2008). Like in our study, the lack of consistent data over a longer period hinders a proper validation in most land use change studies (e.g. Verburg et al. 2008). Therefore, and in order to be consistent with the time frame of observed discharges, historical land use change rates between 1971 and 2006 were determined additionally and revealed that past changes were higher than the projected future scenarios. In spite of this, the risk analyses revealed that in the near future, i.e. up to 2030, the projected land use changes increase flood risk more than climate change (see Fig. 9).

Another important driver for future flood risk is economic development (see Fig. 9) that was considered by adjusted values. This led to a further and clear rise in the assets at risk, particularly in the projected time span (2007–2030) and the strongest urbanisation and economic growth scenarios. Although this approach is state-of-the-art, it has to be emphasised that this part could be further improved. For example, asset appreciation is included by the gross domestic product (GDP), which was used as proxy for the economic growth. However, also asset depreciation should be considered to better account for expenditure of the GDP, e.g. investments for technical improvements. Another drawback arises from the usage of national projections and national historical records of the GDP instead of regional data which may differ notably. It was, however, impossible to get GDP data on the regional scale for the historic as well as the projected time period.

The damage estimation was restricted to damage to residential buildings. Due to the coarse data of the land use scenarios and asset estimation, damage modelling was performed on the meso-scale, not on a building-specific micro-scale. In combination with different specific asset values, 57 variants of damage functions/models were considered. 28 of them, i.e. almost half of the variants, were ruled out since they were incapable of estimating the reported damage of the 2005 flood within a 95 % confidence interval that was created by a bootstrap approach. Only the successfully validated functions were used for assessing potential residential losses of the present and future inundation scenarios. By this, a reduction of the uncertainty bound of flood risk estimates was achieved. Since validation of flood loss models is rarely performed, this aspect clearly distinguishes our study from others.

Finally, all elements of the model chain were combined in a risk analysis. Potential changes in flood risk between the present situation (around 2006) and the near future (around 2030) was assessed. Furthermore, the isolated contribution of the single risk drivers was derived. Altogether, changes in flood risk in the near future, i.e. by 2030, are relatively low compared to changes between 1971 and 2006. Only if strong urbanisation took place associated with economic growth, the assets at risk would increase remarkably. This illustrates that in our study area the effects of climate change on the flood hazard and further on flood risk is slight or negligible compared to the contribution of potential land use changes in the near future, i.e. by 2030. This finding implies that studies on future flood risks should not solely concentrate on climate change as driver. Moreover, stakeholders should carefully watch and govern land use development in flood-prone areas. This is further supported by our investigations of the effects of adaptation options on the flood risk. Adaptation by improving resilience, which was modelled by stronger building restrictions and improved private precaution, could reduce flood risks by 30 %, while ignorance might lead to a further increase of losses by 17 % based on our assumptions.

## Conclusions

Altogether, this study demonstrates that the complex model chain was successfully and consistently established in the study area and could be used 1) to quantify current and future flood risk, 2) to investigate the contributions of each driver to future flood risk, and 3) to estimate effects of non-structural adaptation systematically. Therefore, this study can be used as pilot study for assessing future flood risk and adaptation options at the regional scale.

Ideally, such investigations should be performed in a participatory framework, so that regional and local stakeholders can influence the selection of baseline scenarios and adaptation options. A collaborative modelling platform, as presented by Evers et al. (2012), could facilitate such research.

Although the model chain is regarded to be of good quality and reliability, since a lot of efforts were made to calibrate and validate all elements of the model chain, the overall uncertainty of the model results might still be high, but currently unknown and should be a topic for further research. Nevertheless, this analysis highlights three main aspects: 1) non-structural measures that strengthen private precaution can considerably reduce future flood risks regardless of climate or land use changes and can therefore be regarded and recommended as so-called no-regret measures. Hence, the potential of such non-structural, low cost measures should be better exploited in adaptation strategies and plans than this is currently the case. 2) There is no clear trend of changes in climate or land use on the flood risk. In the study area, future flood risk might be in the same order of magnitude than the current risk, but can also amount to its triple. Therefore, study on future flood risks should consider not only climate change as possible driver, but also land use scenarios and economic development. 3) In any case, ignoring flood risk will lead to increasing losses. Therefore, current efforts on (flood) risk communication and improved private precautionary behaviour need to be enhanced.
